# Regulation of phenylacetic acid uptake is σ^54 ^dependent in *Pseudomonas putida *CA-3

**DOI:** 10.1186/1471-2180-11-229

**Published:** 2011-10-13

**Authors:** Niall D O' Leary, Mark M O' Mahony, Alan DW Dobson

**Affiliations:** 1 Department of Microbiology University College Cork, Cork, Ireland; 2 Environmental Research Institute, University College Cork, Cork, Ireland

## Abstract

**Background:**

Styrene is a toxic and potentially carcinogenic alkenylbenzene used extensively in the polymer processing industry. Significant quantities of contaminated liquid waste are generated annually as a consequence. However, styrene is not a true xenobiotic and microbial pathways for its aerobic assimilation, via an intermediate, phenylacetic acid, have been identified in a diverse range of environmental isolates. The potential for microbial bioremediation of styrene waste has received considerable research attention over the last number of years. As a result the structure, organisation and encoded function of the genes responsible for styrene and phenylacetic acid sensing, uptake and catabolism have been elucidated. However, a limited understanding persists in relation to host specific regulatory molecules which may impart additional control over these pathways. In this study the styrene degrader *Pseudomonas putida *CA-3 was subjected to random mini-Tn*5 *mutagenesis and mutants screened for altered styrene/phenylacetic acid utilisation profiles potentially linked to non-catabolon encoded regulatory influences.

**Results:**

One mutant, D7, capable of growth on styrene, but not on phenylacetic acid, harboured a Tn*5 *insertion in the *rpoN *gene encoding σ54. Complementation of the D7 mutant with the wild type *rpoN *gene restored the ability of this strain to utilise phenylacetic acid as a sole carbon source. Subsequent RT-PCR analyses revealed that a phenylacetate permease, PaaL, was expressed in wild type *P. putida *CA-3 cells utilising styrene or phenylacetic acid, but could not be detected in the disrupted D7 mutant. Expression of plasmid borne *paaL *in mutant D7 was found to fully restore the phenylacetic acid utilisation capacity of the strain to wild type levels. Bioinformatic analysis of the *paaL *promoter from *P. putida *CA-3 revealed two σ^54 ^consensus binding sites in a non-archetypal configuration, with the transcriptional start site being resolved by primer extension analysis. Comparative analyses of genomes encoding phenylacetyl CoA, (PACoA), catabolic operons identified a common association among styrene degradation linked PACoA catabolons in *Pseudomonas *species studied to date.

**Conclusions:**

In summary, this is the first study to report RpoN dependent transcriptional activation of the PACoA catabolon *paaL *gene, encoding a transport protein essential for phenylacetic acid utilisation in *P. putida *CA-3. Bioinformatic analysis is provided to suggest this regulatory link may be common among styrene degrading *Pseudomonads*.

## Background

**Figure 1:**
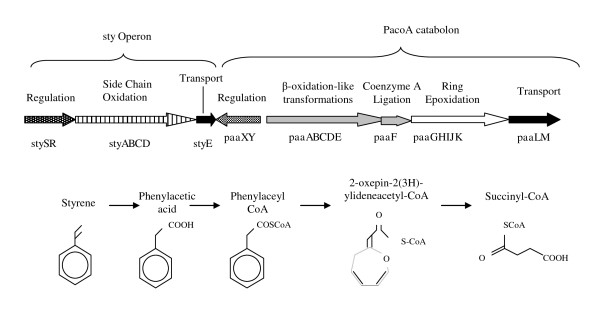
**Over view of styrene catabolism**. Summary schematic of the major steps in styrene and phenylacetic acid degradation. Gene clusters have been grouped broadly in relation to function, while the arrows reflect common operons observed in *Pseudomonads*. However, it should be noted that significant variation in PaCoA catabolon gene organisation is seen in nature, such that a standard consensus schematic is not possible.

Microbial degradation of the major industrial solvent and polymer synthesis monomer styrene has been the focus of intense academic investigation for over 2 decades, most notably in the genus *Pseudomonas*. As a result, a significant body of knowledge has been established regarding the key enzymatic steps as well as the organisation, regulation and taxonomic distribution of the catabolic genes involved [1-4]. In *Pseudomonas *species studied to date, styrene degradation involves an initial "upper pathway", composed of genes encoding the enzymes for styrene catabolism to phenylacetic acid. The upper pathway is regulated by a two component sensor kinase and response regulator system, StySR, which activates transcription of the catabolic genes in response to the presence of styrene, Figure 1, [5-7]. The intermediate, phenylacetic acid, subsequently undergoes an atypical aerobic step of Co-enzyme A activation to yield phenylacetyl CoA (PACoA), which binds to and deactivates a GntR-type negative regulator, PaaX, enabling transcription of the PACoA catabolon. This pathway facilitates the degradation of PACoA to succinyl-CoA and acetyl CoA, Figure 1, [8,9]. The PACoA catabolon was originally identified and characterised in *E. coli *W and *P. putida *U, and has since been found to be widely dispersed among microbial species as one of the four key metabolic routes for microbial, aromatic compound degradation [2,3,10,11]. Thus, while styrene degradation is dependent on the presence of PACoA catabolon genes for complete substrate mineralisation, the PACoA catabolon is commonly identified independently of the *sty *operon genes. Indeed, in *Pseudomonas *sp. Y2 the presence of two, functional PACoA catabolons has been reported, only one of which is linked with the styrene degradation pathway genes. The distinct genetic divergence and gene organisation patterns of these catabolons suggest disparate evolutionary origins, [12]. In relation to the identification and characterisation of styrene linked PACoA catabolons, several strain specific traits have been reported in *Pseudomonas *species studied to date. Comparative analyses of *sty *gene sequences from *Pseudomonas putida *CA-3, *Pseudomonas fluorescens *ST, *Pseudomonas *species Y2 and *Pseudomonas *sp VLB120 reveal a high degree of similarity in terms of percentage identity and structural organisation, [1]. However, functional characterisations in *P. putida *CA-3 and *P. fluorescens *ST have identified different regulatory profiles in relation to catabolite repression inducing carbon sources and nutrient limitation exposure [6,7,13,14]. With respect to the PACoA catabolon, an essential phenylacetic acid uptake mechanism has previously been characterised in *Pseudomonas putida *U, co-ordinately expressed with the catabolic genes [10]. In contrast, a recent proteomic analysis of styrene grown *P. putida *CA-3 cells indicated that phenylacetic acid transport gene products were not detected in styrene grown CA-3, despite the expression of all other PACoA catabolon proteins [15]. Bioinformatic analysis of PACoA catabolon gene organisation in 102 microbial genomes revealed repeated *de novo *clustering of the catabolic genes [3]. However, the authors suggested that recombination events and *in situ *gene replacements by interspecies gene transfer had produced considerable diversity in both gene composition and operonic organisation in the pathways. In light of these findings the question arises as to whether the conserved catabolic function of the PACoA catabolon is subject to varied, host dependent regulatory influences in differing species. Elucidation of such host regulatory influences may identify key flux control points for recombinant strain engineering strategies to optimise biotechnological outputs related to the pathways [9,16-18]. In this study the *Pseudomonas putida *CA-3 genome was randomly mutagenised with a mini-Tn*5 *transposon and isolates screened for altered styrene and phenylacetic acid utilisation phenotypes in an effort to identify key regulatory influences acting on these catabolic pathways in this strain.

## Results and Discussion

### Random Tn*5 *mutant library screening and characterisation

**Figure 2:**
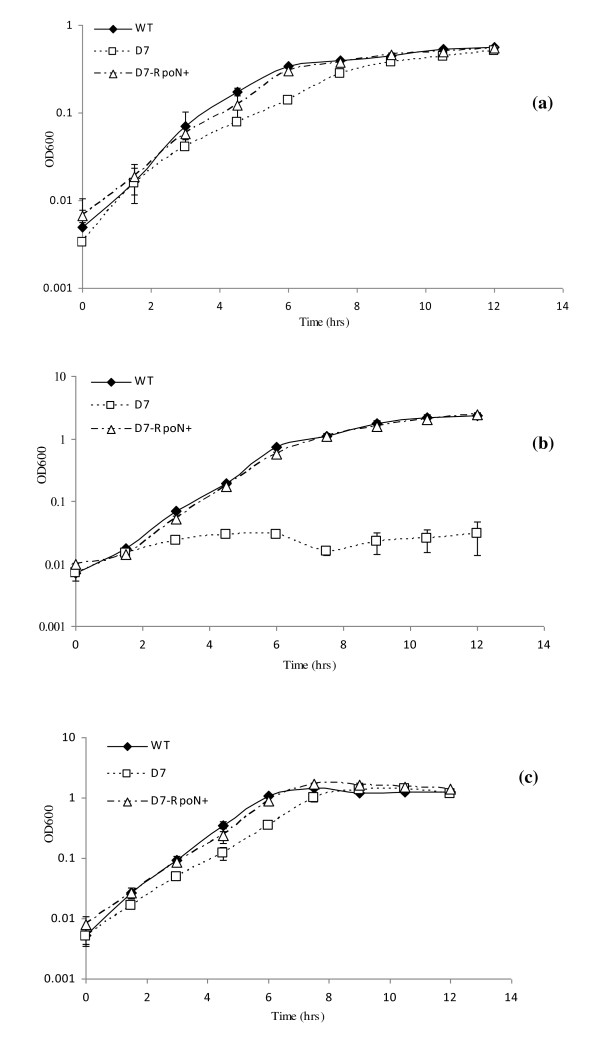
**Growth analyses of wild type and D7 mutant strains**. Growth analyses of *P. putida *CA-3 wild type (WT), *rpoN *disrupted mutant (D7) and RpoN complemented mutant (D7-RpoN+) grown on; (a) styrene, (b) phenylacetic acid and, (c) citrate, respectively.

Approximately 12, 500 transconjugants displaying mini-Tn*5 *dependent kanamycin resistance were generated in the study. Eight of these isolates were found to grow poorly, or not at all, on phenylacetic acid as a sole carbon source in 96 well plates with liquid minimal salts media, (results not shown). Subsequent attempts to cultivate these eight isolates on similar media with styrene as a sole carbon source revealed only one mutant as being capable of growth, D7, achieving wild type biomass levels after a 12 hour period, Figure 2(a). The ability of D7 to grow on styrene indicated that catabolism of the phenylacetic acid intermediate was functional in this mutant. Indeed, subsequent assays of a key enzyme in the process, phenylacetyl-CoA (PACoA) ligase, revealed almost identical activities in styrene grown wild type and D7 mutant cells, (1.8 ± 0.2 and 2.0 ± 0.19 nmol.min^-1^.mg^-1 ^cell dry weight, respectively). However, D7 failed to grow when inoculated into liquid minimal salts media with phenylacetic acid as the sole carbon source, Figure 2(b). The ability of D7 to grow on styrene, (reflecting intracellular phenylacetic acid formation and degradation), but not on extracellular phenylacetic acid as supplied in the media, suggested the potential mini-Tn*5 *disruption of a gene(s) involved in phenylacetic acid uptake. Growth of D7 on a non catabolon related substrate, citrate, produced a similar profile to growth on styrene, Figure 2(a) and 2(c), suggesting core metabolism was intact.

### Identification and complementation of the *rpoN *gene disruption

The insertion site of the mini-Tn*5 *transposon was mapped using two consecutive rounds of arbitrary PCR and the resulting amplicons sequenced and analysed using the GenBank, BLASTn algorithm. The chromosomal region immediately downstream of the Tn*5 *insertion displayed over 98% sequence similarity to *rpoN *genes from other *P. putida *strains, suggesting the gene was disrupted in mutant D7. The nucleotide sequence of the full gene was subsequently generated and submitted to Genbank under the accession number HM756586. In *P. putida *KT2440 the *rpoN *gene forms part of an operon with 4 putative downstream genes encoding members of the phosphotransferase system, including *ptsN *and *ptsO *[19]. While such an operonic structure has not been demonstrated for *P. putida *CA-3, the possibility existed that the observed phenylacetic acid negative phenotype of the D7 mutant may in fact have been as a result of downstream pleiotropic effects of the Tn*5 *insertion in *rpoN*. However, complementation of the disrupted *rpoN *with the cloned, full length wild type gene, (D7-RpoN+), was found to completely restore the strain's ability to grow on styrene and phenylacetic acid, respectively, Figure 2(a) and 2(b). Thus a σ^54 ^deficiency in D7 appeared to be the primary factor responsible for the loss of phenylacetic acid utilisation in this strain. Control experiments with *P. putida *CA-3 wild type and D7 strains carrying the pBBR1MCS-5 expression vector without insert, revealed that the growth profiles presented in Figure 2 were not affected by plasmid maintenance demands or antibiotic presence in the respective media, (results not shown).

### RT-PCR of PACoA catabolon genes in wild type *P. putida *CA-3 and *rpoN *disrupted D7 mutant strains

**Figure 3:**
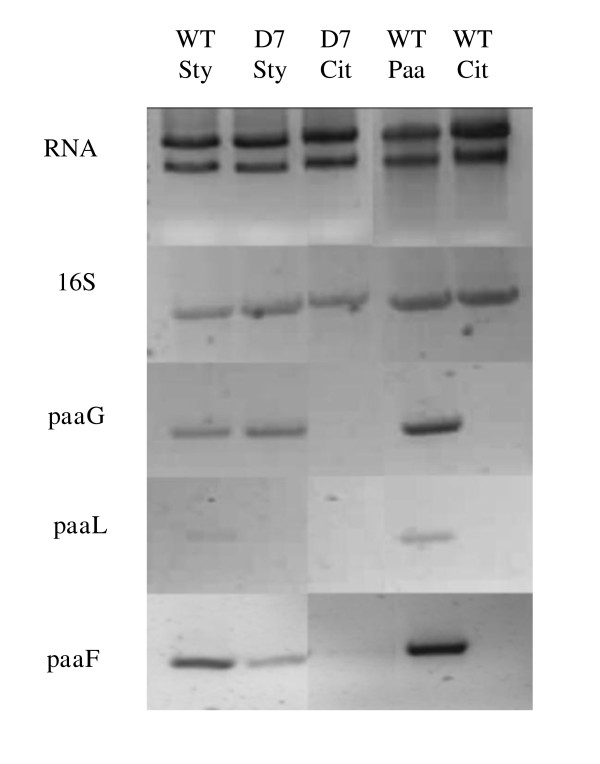
**PaCoA Catabolon gene transcription analyses**. Reverse transcription polymerase chain reaction analysis of *P. putida *CA-3 parent (WT) and *rpoN *disrupted mutant (D7) strains, following growth of cultures on styrene (sty), citrate (cit) and phenylacetic acid (PAA), respectively. 16S rRNA amplification acted as a positive control. The *paaL, paaF *and *paaG*, gene targets (indicated on the left hand side) were selected as representative genes of the operons for phenylacetic uptake, β-oxidation and ring hydroxylation, respectively.

Despite a wealth of available sequence data on the diverse taxonomic distribution and genetic organisation of the PACoA catabolon genes, an extensive review of the existing literature by the authors failed to uncover any prior association between σ^54 ^factors and functional promoters of the PACoA catabolon. Alonso *et al *previously proposed 3 putative operons within the PACoA catabolon in *Pseudomonas sp*. strain Y2, associated with the genes for ring hydroxylation, β-oxidation like conversions and phenylacetic acid transport, respectively [20]. RpoN dependent transcriptional regulation was not proposed in the study. Representative gene targets from these proposed operons were therefore selected for analysis of substrate dependent, transcriptional activation in wild type *P. putida *CA-3 and D7 mutant strains. The target genes selected encoded the PACoA ligase, (*paaF*), an epoxidase subunit 1, (*paaG*), and the phenylacetate permease, (*paaL*). Figure 3 presents a composite image of RT-PCR results, necessitated by the similarity in target gene product sizes. However, the profiles presented accurately reflect those of the individual gels, and take account of variation in contrast levels. Transcriptional activation of the *paaF *and *paaG *genes was readily detected following growth of wild type *P. putida *CA-3 on styrene or phenylacetic acid, while the RT-PCR product for *paaL *was markedly weaker, Figure 3. RT-PCR analysis of D7 mutant strains grown on styrene produced *paaF *and *paaG *transcript profiles similar to wild type cells, however, *paaL *transcripts were not detectable in the mutant, Figure 3. The authors note that Nikodinovic *et al *did not detect the presence of PaaL in a recent proteomic analysis of styrene grown *P. putida *CA-3 cells, [15]. However, the stirred tank reactor growth conditions employed, with continuous feeding of NH_4_Cl to maintain a concentration above 400 mg/L, differed significantly from the batch studies conducted in this investigation. The authors have previously published findings on the significant impact growth conditions can have on the transcriptional regulation of catabolon genes, particularly as inorganic nutrient limitations arise, [21]. It is possible therefore that the low level transcription of paaL reported here during styrene growth may reflect growth conditions not encountered in the proteomic study. 16S rRNA gene RT-PCR indicated equivalent levels of cDNA synthesis in each of the samples. RT-PCR analyses of citrate grown wild type and D7 strains acted as negative controls. To date, the only functional characterisation of phenylacetic acid uptake to have been conducted in *Pseudomonas *was performed with *P. putida *U [10]. In this strain the PaaL permease and PaaM membrane proteins were both reported as essential for phenylacetic acid utilisation and were co-ordinately regulated with transcriptional activation of the other 2 catabolic operons. However, the transcriptional profiling presented in Figure 3, provided preliminary evidence that *paaL *may be differentially regulated in *P. putida *CA-3, in a σ^54 ^dependent manner. The potential for divergent regulatory mechanisms to influence transport in different microbial species is perhaps not surprising however, given that the phenylacetic acid transport system is inconsistently reported in the literature. The *paaM *gene is frequently absent from PACoA catabolons reported in *Pseudomonas *species [12,20,22] while both *paaL *and *paaM *are absent from the PACoA catabolon of *E. coli *W [11]. The authors were unable to identify any *paaM *homologue in *P. putida *CA-3 during this study.

### Over-expression of PaaL in wild type *P. putida *CA-3 and *rpoN *disrupted D7 mutant strains

**Figure 4:**
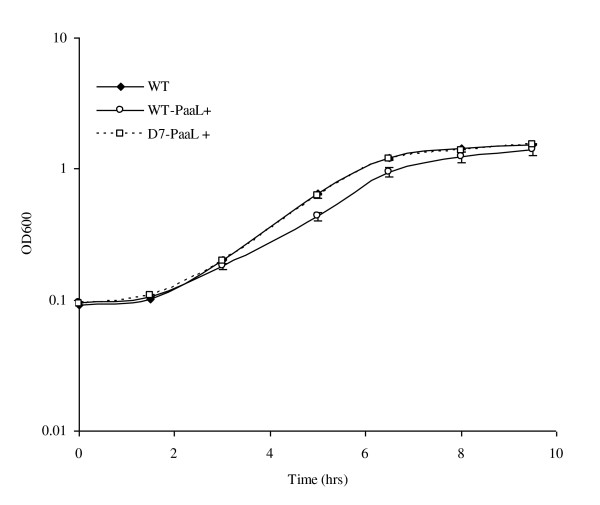
**Effects of PaaL over expression on growth**. Growth on phenylacetic acid of *P. putida *CA-3 wild type (WT) and the wild type and D7 mutant strains harbouring the pBBR1MCS-5 PaaL over expression vector, (WT-PaaL+) and (D7-PaaL+), respectively.

To confirm whether the observed *paaL *gene transcription deficiency was the major contributory factor in the phenylacetic acid negative phenotype of mutant D7, over expression experiments were conducted. The full length 1, 647 kb *paaL *gene was amplified from *P. putida *CA-3 and sequenced, (GenBank accession no: HM638062). The gene was subsequently cloned into the pBBR1MCS-5 expression vector and conjugally transferred into the D7 mutant to give D7-PaaL+. Constitutive expression of PaaL from the pBBR1MCS-5 vector was confirmed by RT-PCR analysis following growth of the host cells on citrate, (result not shown). Growth of D7-PaaL+ on phenylacetic acid was subsequently assessed, with a complete restoration in substrate utilisation by the mutant being observed, Figure 4. Thus, PaaL plays a key role in phenylacetic acid utilisation in *P. putida *CA-3 and *rpoN *dependent regulation appears unique to the transport operon within the PACoA catabolon of this strain. Interestingly, previous work by Jurado *et al *[23] reported that σ^54 ^levels in *P. putida *remain relatively constant throughout growth, ~80 ± 26 molecules per cell, which barely exceeds the number of genome predicted σ^54 ^dependent promoters in *P. putida *KT2440. The possibility existed therefore that limited σ^54 ^availability imparted stringent transcriptional control of the transport mechanism in *P. putida *CA-3, effectively creating a rate limiting step in substrate use. Indeed, previous work by our group demonstrated that over expression of the styrene active transport protein, StyE, in *P. putida *CA-3 resulted in an 8 fold increase in transcriptional activation of the upper pathway [24]. The PaaL expression vector was therefore conjugally transferred into wild type cells to give WT-PaaL+, and growth on phenylacetic acid and PACoA ligase activity assessed. Surprisingly, the observed effect of PaaL over expression in the WT-PaaL+ strain was slower growth on phenylacetic acid compared with the *P. putida *CA-3 parent and D7-PaaL+ strains, Figure 4. In addition, PACoA ligase activity was found to be approximately 22% lower in the WT-PaaL+ strain compared with wild type *P. putida *CA-3 (data not shown). It remains unclear whether the reduced activity observed reflects a direct inhibitory impact on the ligase enzyme, or a general toxicity effect within the cells arising from PaaL over-expression and increased phenylacetic acid uptake. Thus, while PaaL expression is essential for phenylacetic acid utilisation by *P. putida *CA-3, it does not appear to represent a rate limiting step in the process.

### Cloning and bioinformatic analysis of the *paaL *promoter from *P. putida *CA-3

**Figure 5:**
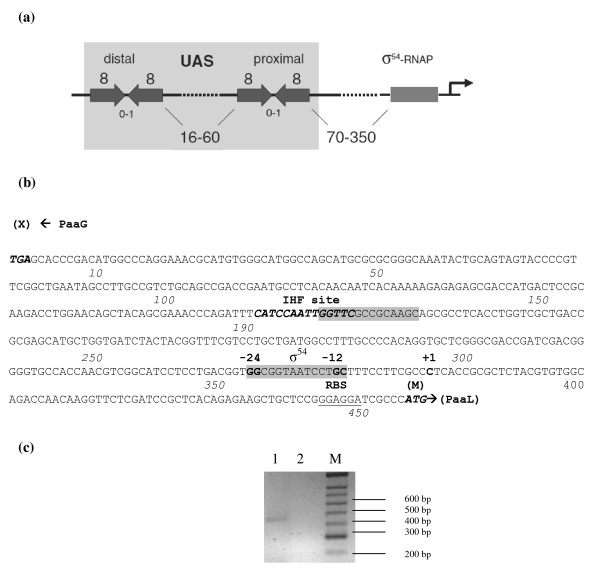
**Analysis of the *paaL *promoter region**. (a) Promoter structure of the archetypal σ^54 ^factor dependent promoter employed by GenomeMatScan to predict the *P. putida *KT2440 sigmulon. The upstream activating sequence UAS is indicated, flanked by distal/proximal enhancer binding protein sites displaying diverse spatial positioning upstream of σ^54^-RNA polymerase promoter complex formation. Schematic originally proposed by Cases *et al*, [38]. (b) Annotated nucleotide sequence of the 456 bp intergenic region between the *paaG *stop codon, (**X**), and the *paaL *start codon **(M) **in *P. putida *CA-3. Nucleotide positions are indicated in italics. An imperfect integration host factor (IHF) binding site is highlighted in bold italics with a tetrameric palindrome indicated by directional arrows. Both consensus GG-N_10_-GC σ^54 ^factor binding sites are highlighted in grey, with the primer extension mapped transcriptional start site indicated numerically (+1). (c) RACE directed RT-PCR amplification of the *paaL *transcriptional start site. Lanes; 1 = 465 bp RACE product, 2 = negative control, (adapter ligated RNA), and M = Hyperladder II DNA marker (Bioline).

The *paaL *promoter region was cloned from *P. putida *CA-3, sequenced and analysed for archetypal σ^54 ^promoter features, Figure 5(a) and 5(b)[19,25]. Analysis of the 458 bp promoter sequence using the search algorithms GenomeMatScan and TRES, failed to identify palindromic or inverted repeat regions, typical of XylR/NtrC family enhancer binding proteins, (EBPs) [19,26]. EBPs are reportedly essential for transcriptional activation of σ^54 ^promoters and facilitate the integration of promoter activation with host signal responses to environmental cues and physiological states, [27,28]. Comparative analysis of the *paaL *promoter with 9 other predicted σ^54 ^promoter sequences from *P. putida *KT2440, was carried out using the Multiple Em for Motif Elucidation algorithm, MEME [29]. The program quantitatively evaluates background noise in similarly regulated promoters to identify the most conserved motifs among them as potential sites for regulator interactions. One highly conserved motif was identified as common to all sequences, which was identified via the TOMTOM motif comparison tool [30] as a σ^54 ^binding site. The site contained the previously reported GG-N_10_-GC,-24/-12 consensus sequence found in all σ^54 ^promoters [25,31]. Virtual FootPrint, which scans for known regulator motifs from the Prodoric database [32] also identified an imperfect integration host factor (IHF) binding site at nucleotide 192, Figure 5(b). However, this site overlaps the MEME predicted σ^54 ^site, prompting the authors to screen for alternative σ^54 ^binding regions. Subsequent analysis of the promoter using the PromScan algorithm, with a cut off score of 0.70, identified a second σ^54 ^consensus site at nucleotide position 356. The proximal location of this site to the proposed *GGAGG *Shine Dalgarno ribosome binding sequence at nucleotide position 455 was more consistent with conventional σ^54 ^promoter architecture, Figure 5(b). Primer extension analysis of RNA extracts from phenylacetic acid grown *P. putida *CA-3 confirmed the transcriptional start site at nucleotide 381, upon sequencing of the 5' RACE PCR product, Figure 5(b) and 5(c).

### Relative sequence identities of *paaL *genes and promoters from diverse *Pseudomonas *species

Clustal W analysis was performed with *paaL *genes and promoters from available PACoA catabolon host genomes, (*P. entomophila *L48, *P. fluorescens *Pf5, *P. putida *F1, *P. putida *KT2440, *P. putida *W619 and *P. putida *GB-1), and styrene degradation associated *paaL *genes from *P. putida *CA-3, Y2 and *P. fluorescens *ST, (Table 1). The analysis revealed greater diversity occurred in promoter sequences than in gene sequences. This is clearly demonstrated among the *paaL *genes from the styrene degraders *P. fluorescens *ST, *P. putida *CA-3 and *Pseudomonas *sp. Y2, which all share > 80% sequence identity with KT2440 *paaL *sequence, but less than 16% identity at the respective promoter level, Table 1. Among the three styrene degrading strains the authors note that the *paaL *promoters are 100% identical, while the catabolic genes share ~97% sequence identity, Table 1.

**Table 1:** Clustal W alignment of microbial *paaL *genes and promoters.

Percentage Sequence Identity										
-	**CA-3**	**F1**	**GB1**	**KT2440**	**L48**	**Pf5**	**ST**	**W619**	**Y2**	***paaL*** **Genes**
**CA-3**	-	81.1	80.9	81	82.7	81.5	98.4	69.3	97.2	**CA-3**
**F1**	15.6	-	92.5	98.3	87.4	83	81.9	72	81.8	**F1**
**GB1**	14.3	78.7	-	92.5	87.9	83.4	81.3	73.1	81.5	**GB1**
**KT2440**	15.6	99.3	79.4	-	87.7	83	81.7	72.1	81.6	**KT2440**
**L48**	14.3	27	24.9	27	-	85.6	82.9	73.1	83.2	**L48**
**Pf5**	19.5	18.6	39.8	18.6	38.9	-	81.5	70.2	81.8	**Pf5**
**ST**	100	15.6	12.9	15.6	14.8	20.4	-	69.8	96.6	**ST**
**W619**	23.5	58.8	60	58.8	45.9	23.5	27.1	-	70.2	**W619**
**Y2**	100	15.6	11.8	15.6	11.7	20.4	100	27.1	-	**Y2**
***paaL*** **Promoters**	**CA-3**	**F1**	**GB1**	**KT2440**	**L48**	**Pf5**	**ST**	**W619**	**Y2**	-

ClustalW alignment generated percentage sequence identities of *paaL *genes (top section) and respective promoters (bottom section) from a number of *Pseudomonas *species harbouring the PaCoA catabolon. CA-3, F1, GB-1, KT2440, ST, W619 and Y2 represent individual *P. putida *strains. The Pf5 and ST strains are members of the *P. fluorescens *group while L48 represents *P. entomophila *L48.

## Conclusions

To our knowledge this is the first study to report σ^54 ^dependent regulation of PaaL expression in phenylacetic acid utilisation by a *Pseudomonas *species. Since other groups have previously suggested σ^70 ^dependent regulation of the transport system, [5,10,12,20] we questioned whether such regulation might be unique to *P. putida *CA-3, or have a potentially broader significance in the field of styrene/phenylacetic acid microbial catabolism. Our analyses of the genetic diversity of *paaL *genes and promoters suggest that a relatively recent recombination event involving *de novo *clustering of *paa *genes [3] with the *sty *operon may have occurred. In this scenario, incorporation of the σ^54 ^dependent regulation of *paaL *may have been an arbitrary event, following the "black cat/white cat" random promoter association model proposed by Cases and de Lorenzo in relation to novel catabolic pathways [33]. However, irrespective of the origins of σ^54 ^regulation of *paaL*, the identical promoter structures suggest that biotechnological applications targeting this pathway should consider the potential for a functional role of σ^54 ^dependent regulation in phenylacetic acid assimilation by these strains.

## Methods

### Bacterial strains, plasmids and growth conditions

*P. putida *CA-3 is a styrene degrading, bioreactor isolate previously characterised by our group [14]. Cultures were maintained on LB agar for use in overnight inoculations into cultivation media. *P. putida *CA-3 was routinely grown in 100 ml of liquid minimal salt media in 1 L flasks at 30°C, shaking at 120 rpm. The basal salts media contained 7.0 g K_2_HPO_4_, 3.0 g KH_2_PO_4_, 1.0 g (NH_4_)_2_SO_4 _per litre distilled water, and 2 ml of 1 M MgSO_4 _added post autoclaving. Carbon sources were added to the following concentrations; 15 mM phenylacetic acid and 10 mM citrate. Growth on styrene required substrate provision in the gaseous phase via addition of 70 μl of liquid styrene to a test tube fixed centrally to the bottom of a baffled 1 L Erlenmeyer flask [6]. Cell growth was monitored by measuring optical density at 540 nm. *E. coli *CC118λ*pir *hosted the mini-Tn*5 *derivative pUTKm1 [34]. The suicide plasmid has the R6K origin of replication and encodes resistance to kanamycin and ampicillin. HB101 (pRK600) was used as a helper in triparental mating experiments, providing both resistance to chloramphenicol and the *tra *function for pUTKm1 mobilization [34]. PCR2.1-TOPO vector was used to clone polymerase chain reaction (pcr) amplification products and transformations performed with One shot^® ^Top10F' competent *E. coli *cells, (Invitrogen, California). *E. coli *strains were grown on Luria Burtani medium at 37°C. Host/plasmid associations were maintained during growth via the incorporation of appropriate antibiotics to media at the following concentrations; 100 μg/ml ampicillin, 25 μg/ml chloramphenicol, 50 μg/ml kanamycin and 20 μg/ml gentamycin.

### Nucleic acid manipulations

Genomic DNA isolation was performed according to Ausubel *et al. *[35]. Plasmid DNA was isolated from *E. coli *using a plasmid Miniprep Kit (Qiagen), as per manufacturer's instructions. DNA visualisations were performed via 1% agarose gel electrophoresis in standard TE buffer followed by EtBr staining and photographic capture in a GeneWizard UV trans-illuminator/gel documentation system, (Syngene Bio Imaging). Oligonucleotide primers used in this study were synthesized by Sigma-Genosys, Ltd. (United Kingdom), and are listed in Table 2. Nucleic acid sequencing was performed by GATC Biotech AG, (Germany), using ABI 3730 × l technology. Routine polymerase chain reactions were carried out in a PTC-200 thermal cycler (MJ Research) using Taq DNA polymerase (Fermentas). High-fidelity amplification requirements were performed with proof-reading, VentR^® ^DNA polymerase (NEB).

**Table 2:** Primers for PCR amplifications.

Primer	Sequence 5'-3'	Annealing temp°C
GS326	acgatgcccagggagtagaga	60
OP2-55	gctgatggcgatgaatgaaca	55
TNInt2	cctgcaggcatgcaagcttcggc	65
27F	agagtttgatcatggctcag	55
1492R	ggttaccttgttacgactt	55
paaFf paaFr paaGf	ggttgagcatgtaggacggt gccaataccgccttgcttga ccgaaggcaactgggtcac	57 57 55
paaGr	aggcggcgttcttgttctg	55
paaLf	cggcatgctcgcgaccacctg	60
paaLr	aaagcgatgttctgcgactc	60
Sig54f-*Hin*d	tattac*aagctt*atgaaaccatcgctgtcctaaaaatg^a^	60
Sig54r-*Xba*	atcatt*tctaga*ctacatcagtcgcttgcgttcgctcga^b^	60
paaLproF	gccgcgcaacagccagagc	63
paaLproR	cgccgagatgccgaggaagg	63
paaLf-*Hin*d	tattac*aagctt*atgacagccctgcgctccttcacctt^a^	60
paaLr-*Xba*	atcatt*tctaga*ctagtggttactggccttggct^b^	60

a: Hind III restriction site, b: Xba I restriction site.

Oligonucleotide sequences and annealing temperatures utilised in polymerase chain reaction amplification of gene targets from *P. putida *CA-3 in this study.

### Enzyme assays

Styrene monooxygenase activity was assessed colorimetrically using whole cell transformations of indole to indigo as previously described [36]. PACoA ligase activity was measured via the method of Martinez-Blanco *et al *[37]. Activities are expressed as nmol product formed min^-1 ^(mg cell dry weight)^-1 ^for both assays. Cells were harvested at mid-exponential phase unless otherwise stated.

### Random mini-Tn*5 *mutagenesis

A triparental mating approach was used to introduce pUTKm1 into *P. putida *CA-3, as previously described [9]. The mating reaction was plated out on minimal salts media containing 10 mM citrate and 50 μg/ml kanamycin to select for *P. putida *CA-3 transconjugants harbouring successful, mini-Tn*5 *genomic insertions. 12, 500 transconjugants were screened for transposition events that disrupted phenylacetic acid metabolism on solid minimal media containing 15 mM phenylacetic acid and kanamycin 50. Transconjugants which failed to grow on phenylacetic acid were subsequently screened for an ability to utilise styrene as a sole carbon source.

### Mapping of transposon insertion sites

Arbitrarily primed PCR was employed to map the gene disruption sites utilising previously published oligonucleotide sequences and appropriate thermal cycling parameters [38]. Products were visualised on 1% agarose gels, purified using a QIAEX II Gel extraction kit and sequenced using the mini-Tn*5 *internal primer, TNInt2 (Table 2).

### RT-PCR analyses

RNA was isolated from *P. putida *CA-3 using a Qiagen RNeasy^® ^Mini Kit, as per the manufacturer's instructions. The purified RNA was treated with TURBO DNA-free™ DNase kit, (Ambion), to ensure complete removal of DNA. All RNA samples were routinely subjected to 16S rRNA gene PCR to confirm the absence of DNA contamination. Reverse transcription was performed with 1 μg of total RNA using random hexamer priming, 1 mM dNTPs, 10 U Transcriptor reverse transcriptase with 1× reaction buffer, (Roche), and SUPERNaseIn (Ambion) in a 20 μl reaction volume. Reactions were incubated at 25°C for 10 minutes, followed by 30 minutes at 55°C. 2 μl of the respective RT reactions were employed as template in subsequent PCR reactions. Amplification of the 16S rRNA gene acted as positive control for RT-PCR analyses (universal primers 27f, 1429r), while the following pathway operon specific targets were selected for transcriptional profiling; *paaF *encoding PACoA ligase, *paaG *encoding a member of the ring hydroxylation complex, and the *paaL *encoding phenylacetate permease. Oligonucleotide sequences for the respective gene targets are provided in Table 2.

### Complementation of the RpoN disrupted mutant

Available nucleotide sequences of *rpoN *genes from *P. putida *species were retrieved from the GenBank database and used to construct degenerate primers for the amplification of *rpoN *from *P. putida *CA-3. Restriction sites were mis-primed into the oligonucleotides, (Sig54f-*Hin*d and Sig54r-Xba, respectively), to allow directional cloning into the pBBR1MCS-5 expression vector enabling *lac *promoter expression [39]. Amplification of the desired *rpoN *target was confirmed by sequencing, prior to enzymatic restriction and ligation using standard conditions (GenBank accession no. HM756586). Transformations were carried out with Top 10F' competent *E. coli *cells, (Invitrogen, California), in accordance with the manufacturer's instructions. The pBBR1MCS-5 vector facilitated blue/white colony screening on LB-IPTG-β-gal-Gent20 medium to identify successful cloning events, which were confirmed by culturing, plasmid isolation and restriction with *Hin*dIII and *Xba*I. Conjugal transfer of this RpoN expression vector into *P. putida *CA-3 D7 (carrying a Tn*5*::*rpoN *gene disruption), was performed by tri-parental mating with the Top 10F' *E. coli *host and the HB101(pRK600) helper, as previously described. *P. putida *CA-3 D7 transconjugants were isolated from the mating mix by spread plating 50 μl aliquots onto minimal salts media containing10 mM citrate and 20 μg/ml gentamycin. The pBBR1MCS-5 vector, (lacking any insert), was also transferred into *P. putida *CA-3 wild type and D7 mutant strains to provide controls for subsequent growth studies. All growth curves were conducted in triplicate.

### Cloning and over expression of the phenylacetate permease, PaaL

Degenerate *paaL *primers, harbouring similar mis-primed restriction enzyme sites as before (paaLf-*Hin*d & paaLr-*Xba*, Table 2), were designed based on sequence data from *P. fluorescens *ST and *Pseudomonas *sp. Y2, [20,22]. Cloning, screening and vector/insert confirmation in the Top 10F' *E. coli *host was conducted as described previously. Tri-parental mating to achieve conjugal transfer of the vector into *rpoN *disrupted *P. putida *CA-3 cells was also performed as before. Transconjugants were subsequently screened for any restoration of the ability to grow in minimal salts media with phenylacetic acid as the sole carbon source. To determine whether strict regulation of PaaL expression represented a rate limiting feature of extracellular phenylacetic acid utilisation in wild type *P. putida *CA-3, the PaaL expression vector was also conjugally transferred into the parent strain. RT-PCR analysis was employed to confirm constitutive expression of PaaL from the vector under non inducing growth on minimal salts citrate. Over expression strains were subsequently grown in minimal salts media with phenylacetic acid to facilitate growth profiling and PACoA ligase activity determination. All growth curves were conducted in triplicate. It should be noted that a degenerate pcr strategy was employed to screen the *P. putida *CA-3 genome for a *paaM *permease gene homologue, but none was detected.

### Isolation and analysis of the *paaL *promoter

Primers were designed to amplify the promoter region of the *paaL *gene based on the sequence data of the PACoA catabolon of *Pseudomonas *sp. strain Y2. The primer set (paaLproF and paaLproR, Table 2), amplified a 964 base pair region spanning the 3' end of the *paaG *gene, the intergenic region and the 5' end of *paaL*. The complete *paaL *gene and promoter region have been submitted to GenBank, (Accession number HM638062). A number of putative σ^54 ^dependent promoters of transport proteins from the *P. putida *KT2440 genome, [19], were comparatively analysed with the CA-3 *paaL *promoter using the MEME software suite and the TOMTOM motif comparison tool to identify any highly conserved motifs, [29,30]. 5'RACE primer extension analysis (Ambion) was also carried out to map the *paaL *transcriptional start site, as per the manufacturer's instructions. In brief, this approach involved the generation of 5' adapter ligated RNA, reverse transcription with random decamers and PCR amplification from cDNA using 5' adapter specific and 3' gene specific primers, OP2-55 and GS-441 (Table 2). The PCR thermal cycling conditions included a 5 min hot start at 94°C, followed by 45 cycles of 94°C × 60 s, 55°C × 45 s and 72°C × 30 s.

## Authors' contributions

NOL and AD contributed to the experimental design. NOL and MOM conducted the research. NOL prepared the manuscript. All authors have read and approved the manuscript.
